# Metformin Alleviated A*β*-Induced Apoptosis via the Suppression of JNK MAPK Signaling Pathway in Cultured Hippocampal Neurons

**DOI:** 10.1155/2016/1421430

**Published:** 2016-06-15

**Authors:** Bin Chen, Ying Teng, Xingguang Zhang, Xiaofeng Lv, Yanling Yin

**Affiliations:** ^1^Department of Endocrinology and Metabolism, General Hospital of Beijing Military Region, Beijing 100700, China; ^2^Affiliated Hospital of Beijing Institute of Aeronautical Material, Beijing 100060, China; ^3^Department of Neurobiology and Beijing Institute for Brain Disorders, Capital Medical University, Beijing 100069, China

## Abstract

Both diabetes and hyperinsulinemia are confirmed risk factors for Alzheimer's disease. Some researchers proposed that antidiabetic drugs may be used as disease-modifying therapies, such as metformin and thiazolidinediones, although more evidence was poorly supported. The aim of the current study is to investigate the role of metformin in A*β*-induced cytotoxicity and explore the underlying mechanisms. First, the experimental results show that metformin salvaged the neurons exposed to A*β* in a concentration-dependent manner with MTT and LDH assay. Further, the phosphorylation levels of JNK, ERK1/2, and p38 MAPK were measured with western blot analysis. It was investigated that A*β* increased phospho-JNK significantly but had no effect on phospho-p38 MAPK and phospho-ERK1/2. Metformin decreased hyperphosphorylated JNK induced by A*β*; however, the protection of metformin against A*β* was blocked when anisomycin, the activator of JNK, was added to the medium, indicating that metformin performed its protection against A*β* in a JNK-dependent way. In addition, it was observed that metformin protected the neurons via the suppression of apoptosis. Taken together, our findings demonstrate that metformin may have a positive effect on A*β*-induced cytotoxicity, which provides a preclinical strategy against AD for elders with diabetes.

## 1. Introduction

In the world, over 35 million people are suffering from Alzheimer's disease (AD), and the number of patients is estimated to be more than 100 million by 2050 [1]. Unfortunately, no medications have been testified to be effective for AD by the US Food and Drug Administration to date [2]. As an irreversible neurodegenerative disease, the research hotspot for AD has turned to its preclinical stage [3]. By now, AD is featured by progressive memory loss and a gradual impairment in cognitive function, finally resulting in premature death of the individual usually during 3–9 years after diagnosis [4, 5]. The neuropathological characteristics of AD include the existence of extracellular senile plaques with amyloid-*β* (A*β*) protein, neurofibrillary tangles with intracellular and abnormally phosphorylated tau protein, and a sharp loss of neurons and synapses [6–8]. Based on the confirmed pathological changes, the “amyloid cascade hypothesis” is most acceptable view currently. Therefore, strategies to decrease A*β* production, increase A*β* removal, or reduce A*β*-induced cytotoxicity are considered as effective to Alzheimer's disease.

Nowadays, the scientists have laid more and more emphasis on the relationship between AD and diabetes, and diabetes has absolutely been considered as a risk factor for AD [9]. Type 2 diabetes was one of the strongest risk factors of AD, which increases the risk level to double compared with people without this disease [10, 11]. Metformin (N′,N′-dimethylbiguanide) is acceptably used as the first-line medicine for the therapy of type 2 diabetes [12]. Metabolic dysfunction is one of the vital pathological features of AD, too. Moreover, a new study suggests the balance of metabolism ameliorates cognitive function. Metformin is one of FDA approved drugs, which is potentially applied in alleviating metabolic dysfunction in AD [13]. As an effective medication aimed at AD and diabetes both, metformin deserves a further exploration.

The trials of metformin on AD treatment have been applied and testified to be successful. Hettich et al. reported that metformin decreased the hyperphosphorylation of TAU protein, BACE1 protein expression, and the production of A*β* peptides both* in vivo* and* in vitro*; thereafter metformin will be valuable for therapy against AD via interfering with both pathological hallmarks of AD [14]. However, Picone et al. reported metformin increased APP and presenilin levels, at the same time, induced oxidative stress, mitochondrial damage, and cytochrome C release, which resulted in an aggregation of AD [15]. The paradoxical results may be caused by different experimental results, but due to the large population suffering from both AD and diabetes, further exploration should be carried out, so as the mechanism of metformin. In the current study, we set up a cytotoxicity cellular model with A*β* exposure and observed the effect of metformin on it. Strikingly, we confirmed the therapeutic value of metformin on A*β*-induced cytotoxicity, suggesting metformin may be a promising agent of clinical treatment for both AD and diabetes.

## 2. Method

### 2.1. Hippocampal Neurons Culture and Treatments

Pregnant Wistar rats were bought from the animal facility of the Peking University. All experimental animals were approved by the Institutional Animal Care and Use Committee (IACUC). Hippocampal neurons were cultured as the previous method [16]. The neurons were used for experiments on about 10 days after being plated. For the A*β* incubation studies, freshly diluted oligomer A*β*1–42 (Sigma-Aldrich) was added to the hippocampal cultures, and electrophysiological measurements were performed after 24 h.

### 2.2. Thiazolyl Blue Tetrazolium Bromide (MTT) Assay

The cell viability of cultured neurons was evaluated with a MTT assay. Neurons cultured in 96-well plates were incubated with MTT solution at 37°C for 4 h. Then, dimethyl sulfoxide (DMSO, 100 *μ*L/well) was used to dissolve the formazan crystals; cell viability was measured with the percentage of MTT decrease compared with that under the control conditions.

### 2.3. Lactate Dehydrogenase (LDH) Release Assay for Cell Death

Cell death was measured by LDH leakage. The medium of the cultured neurons in 6-well plates was gathered to evaluate the LDH release with an LDH assay kit by the manufacturer's instructions.

### 2.4. JC-10 Staining

The mitochondrial membrane potential is one of the hallmarks of apoptosis, which is detected with JC-10 mitochondrial membrane potential assay kit (AAT Bioquest, Sunnyvale, USA). The medium was withdrawn and the neurons were exposed under JC-10 for 30 min at 37°C in an incubator. These neurons were rinsed twice with ice-cold PBS, and then the fluorescence intensity of JC10 was observed via fluorescence microscopy. Decreased fluorescence of the neurons was considered as the mitochondrial membrane potential collapsed.

### 2.5. Western Blot Analysis

To determine the phosphorylation level of ERK1/2, JNK and p38, whole-cell extracts were got and fractionated with SDS-PAGE. After electrophoresis, we electrotransferred the proteins onto the nitrocellulose membranes, and blotted them with primary antibody for phospho-Thr202/Tyr204 ERK1/2 (Cell Signaling Technology, CST, Danvers, MA, USA), ERK1/2 (CST), phospho-Thr183/Tyr185 JNK (CST), JNK (CST), phospho-Thr180/Tyr182 p38 (Abcam Technology, Cambridge, MA, USA), p38 (Abcam), and the corresponding secondary antibodies. The Enhanced Chemiluminescence (ECL) kit (GE Healthcare, UK) was selected to represent the signals.

### 2.6. Statistical Analysis

Data are presented as mean ± SE. Statistical analysis is performed by one-way analysis of variance and followed by all pairwise multiple comparison procedures with Bonferroni test (sigmastat 10.0). *p* < 0.05 is confirmed to be statistically significant.

## 3. Results

### 3.1. Metformin Alleviates A*β*-Induced Cytotoxicity in a Concentration-Dependent Manner

**Figure 1:**
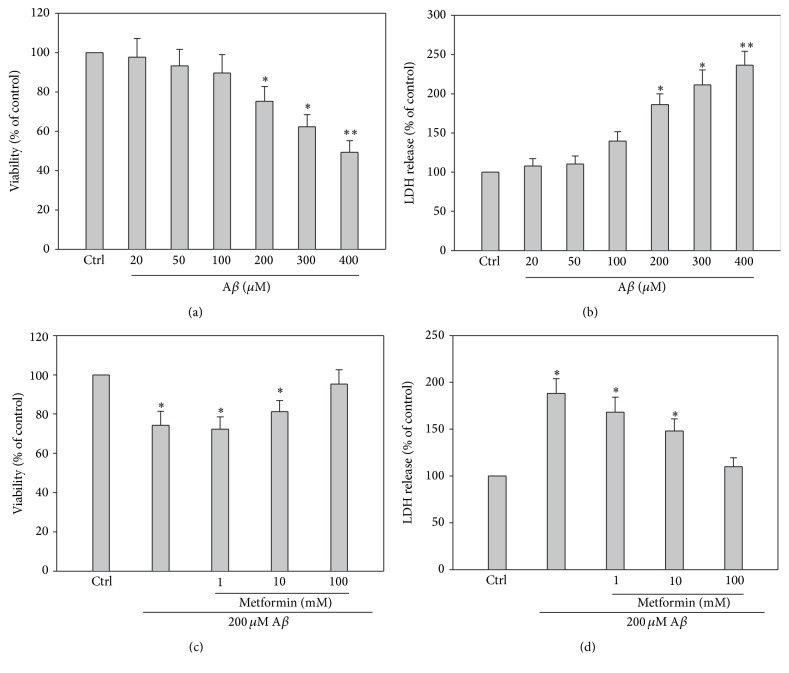
Concentration-dependent cytotoxicity of cultured hippocampal neurons induced by exposure under different A*β* concentrations from 20 to 400 *μ*M. Metformin alleviated A*β*-induced cytotoxicity also in a dose-dependent manner. (a) and (b) Quantitative analysis of MTT and LDH release assay showed that A*β* decreased cell viability from 50 to 200 *μ*M (^*∗*^ *p* < 0.05, ^*∗∗*^ *p* < 0.01 versus that of control, *n* = 6 per group). (c) and (d) Quantitative analysis of MTT and LDH release assay represented that metformin pretreatment decreased injuries induced by 200 *μ*M A*β* in a concentration-dependent way. As shown in (c) and (d), 100 mM metformin can increase the neuronal viability distinctively.

Hippocampal neurons were injured by A*β* at different concentrations for 24 hours. Neuronal death was increased after A*β* exposure compared with that of the control group at concentrations ranging from 20 to 400 *μ*M A*β* using MTT and LDH assay (Figures 1(a) and 1(b)). The neuronal viability was decreased from the dose of 200 *μ*M (^*∗*^ *p* < 0.05, *n* = 6), and when A*β* dose was increased to over 200 *μ*M, the viability decreased significantly (^*∗*^ *p* < 0.01, *n* = 6). Considering we are not sure of the role of metformin in A*β*-induced cytotoxicity, 200 *μ*M A*β* was chosen to set up the cellular model. Although metformin is testified to play a role in AD, its tone and mechanism are poorly known and required to be explored. We addressed the viability and LDH release of the hippocampal neurons treated with naive A*β* 200 *μ*M or metformin treatment at different concentrations. At this dose of 100 mM, metformin suppressed the neuronal injuries by 200 *μ*M A*β* (*p* < 0.05, *n* = 6), while the effect of 1 or 10 mM metformin against A*β*-induced injuries was not distinct (Figures 1(c) and 1(d)), which suggested that the protective effect of metformin was dose-dependent.

### 3.2. Metformin Reversed A*β*-Induced Hyperphosphorylation of JNK in the Hippocampal Neurons

**Figure 2:**
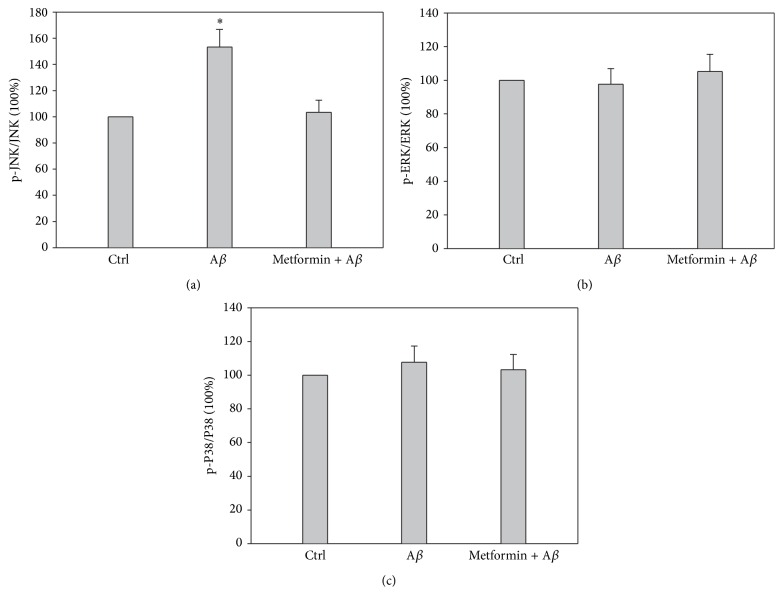
Metformin rescued A*β*- (200 *μ*M) induced hyperphosphorylation of MAPK JNK. (a), (b), and (c) Quantitative analysis of western blot assay showed that A*β* increased the phosphorylation level of JNK, which was reserved by metformin (^*∗*^ *p* < 0.05, *n* = 5), while there was no significant difference in the phosphorylation level of ERK1/2 or P38 between the experimental groups (*p* > 0.05, *n* = 5).

As we observed, metformin salvaged the neurons exposed to A*β*; however, the mechanism was still obscure and required to be explored further. Mitogen-activated protein kinases (MAPK) are an evolutionarily conserved signal-transduction family, in which the serine/threonine kinases are unique to eukaryotes. JNK is an important member of this family and involved in numerous stimuli, including excitotoxic stress and DNA damage [17]. Moreover, it has been reported that the activation of JNK will always result in the proapoptotic signaling activation by inhibiting the antiapoptotic proteins, such as Bcl-xL and Bcl2 [18]. Recently, JNK signaling pathway has been considered as “a therapeutic target for Alzheimer's disease” because of its vital role in etiology analysis of Alzheimer's disease [19]. Increased phosphorylated JNK (pJNK) and a positive colocalization with A*β* were revealed in human postmortem brain samples of AD patients [20, 21]. In addition, the activation of JNK was reported to be positively related with cognitive decline, a marker of AD [22]. To determine via which signaling pathway metformin performs its protection against A*β* exposure, we immunoprecipitated ERK1/2, JNK, and P38 and used western blotting to evaluate their activation with the phosphorylation levels. There was a significant difference only in the phosphorylation level of JNK between the experimental groups under naive A*β* exposure (*p* < 0.05, *n* = 5). The results indicated that A*β* exposure increased the phosphorylation of JNK but not ERK1/2 or p38 (Figures 2(a), 2(b), and 2(c); the presentative blots are not shown in this paper). Further, when metformin was added before A*β* exposure, the hyperphosphorylation of JNK was blocked, suggesting JNK played a vital role in A*β*-induced cytotoxicity and metformin-involved protection.

### 3.3. Metformin Decreased A*β*-Induced Cytotoxicity in a JNK-Dependent Way

**Figure 3:**
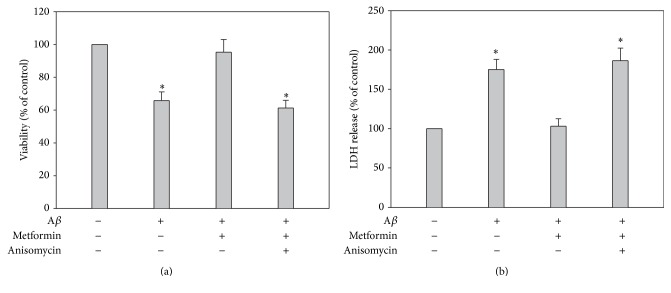
Metformin salvaged A*β*-induced cytotoxicity via MAPK JNK signaling pathway. (a) and (b) Quantitative analysis of MTT and LDH represented that A*β* increased the phosphorylation of JNK, which could be reserved by metformin treatment (^*∗*^ *p* < 0.05, *n* = 5). In addition, anisomycin treatment blocked metformin-involved protection, suggesting the depression of JNK had a vital role in the effect of metformin.

Although we investigated the role of metformin against A*β*-induced cytotoxicity via JNK signaling pathway, it still needs to elucidate the key role of JNK, which may be an indicator of strategic target. Metformin has been reported to play its role via a lot of signaling pathways. We added anisomycin, the activator of JNK, to the medium, to investigate the protection of metformin under this condition. Interestingly, metformin failed to protect hippocampal neurons with the interference of anisomycin (Figures 3(a) and 3(b)). Based on this result, we conclude that JNK-involved signaling pathway is the key target of metformin against A*β*-induced cytotoxicity, although we still cannot decide which stage of AD 200 *μ*M A*β* is corresponding to by now.

### 3.4. Metformin Decreased A*β*-Induced Cytotoxicity through the Inhibition of Apoptosis

**Figure 4:**
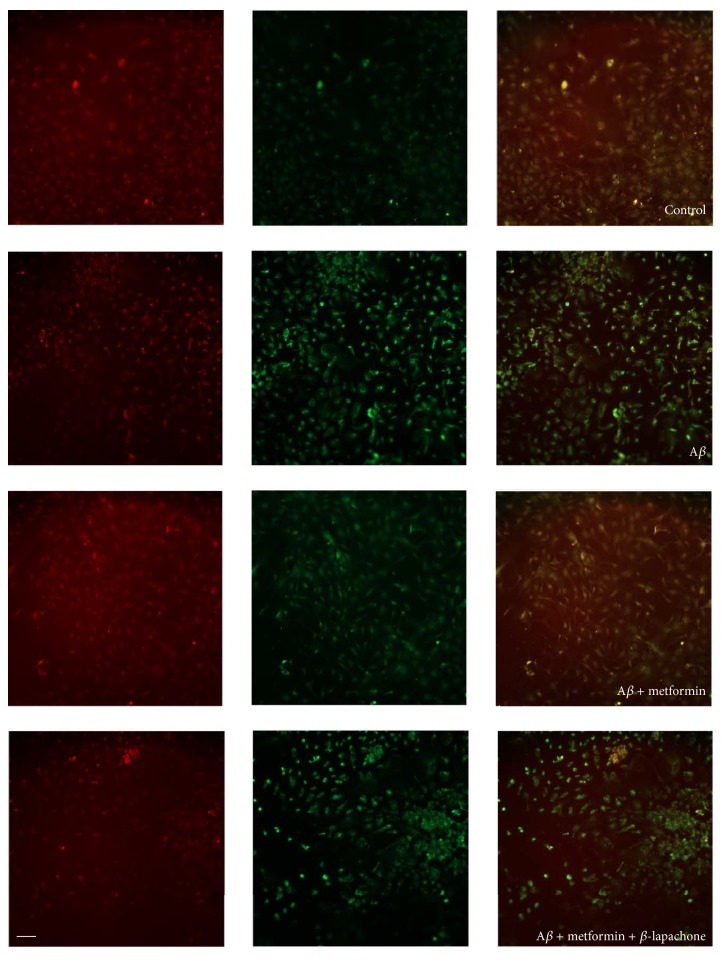
Metformin decreased A*β*-induced cytotoxicity by suppression of apoptosis. Representative results of JC-10 staining. Quantitative analysis of fluorescence intensity showed that A*β* increased the phosphorylation level of JNK, which was reserved by metformin (^*∗*^ *p* < 0.05, *n* = 5). However, when *β*-lapachone, the activator of apoptosis, was added into the medium, the metformin-involved protection was blocked, indicating metformin played its role via decreasing apoptosis; scale bars, 50 *μ*m.

To date, three kinds of programmed cell death have been confirmed according to the morphological criteria, including apoptotic cell death (type I), autophagic cell death (type II), and necrosis or cytoplasmic cell death (type III). Based on the correlation between JNK and apoptotic cell death, we further observed the mitochondrial membrane potential of the cultured neurons with JC-10 staining, which is an acceptable indicator of apoptosis. The statistical analysis results showed that A*β* increased the apoptosis of cultured hippocampal neurons, which was reversed by metformin (*p* < 0.05, *n* = 3, Figure 4; the statistical results are not shown in this paper). When *β*-lapachone, the activator of apoptosis, was added to the medium, the protective effect of metformin was blocked, suggesting metformin performed its influence by decreasing the cellular apoptosis. Taken together, JNK-involved signaling pathway contributes to 200 *μ*M A*β*-induced cytotoxicity, which can be reversed by metformin treatment.

## 4. Discussion

As a devastating neurodegenerative disorder, emphasis is laid on Alzheimer's disease by worldwide scientists in both prevention and treatment strategies. In the past years, many factors including aging, genetic, and environmental ones have been confirmed to contribute to the development and progression of AD. Although drugs for AD have been clinically tested, the therapeutic effects are poor by now, suggesting further researches are still required. Epidemiological studies intensely indicate that metabolic defects result in the functional modifications involved in cerebral aging and in AD pathogenesis. The dysfunction of cerebral glucose metabolism in early stages of AD is testified [23], and the molecular markers of insulin resistance is found to colocalize with tau inclusions in AD brain [24], indicating that the insulin-involved pathway may be a vital element to the AD pathophysiological cascade [25].

More and more evidence provides that diabetes mellitus (DM) is more than a common syndrome, which is considered as a risk factor for AD in the elderly. The high prevalence of DM and AD in the elderly population and their close correlation urgently call for a proper concomitant pharmacotherapy according to FDA approval. Metformin is a biguanide which has multipurpose influences on metabolism, by increasing insulin-sensitization, glucose uptake and the activation of AMP activated protein kinase, and so on. To date, the effect of metformin is focused on in the strategy for both DM and AD. Although more and more attentions have been paid to the usage of metformin in AD treatment, its tone and mechanism are still controversial and required to be explored further.

Many trials have been performed with metformin treatment on AD, although its effects are still controversial. Chen et al. reported that metformin increased A*β* efflux across the blood-brain barrier under diabetic context; then hippocampal A*β*1–40 or A*β*1–42 and neuronal apoptosis were significantly decreased, which ameliorated memory impairment [26]. Hettich et al. reported that metformin decreased the phosphorylation of tau at AD-relevant phosphosites and the protein expression of beta-secretase (BACE1) by interfering with an mRNA-protein complex [14]. However, some other scientists supported different insights about the influence of metformin on AD treatment. Picone et al. addressed that metformin activated NF-*κ*B by translocating it from the cytoplasm to the nucleus where NF-*κ*B increased APP and Pres 1 transcription, suggesting metformin promoted the progression of AD [15]. Chen et al. found that metformin significantly increased the production of both intracellular and extracellular A*β* species via the upregulation of BACE1 [27]. The paradoxical results may result from different experimental conditions, such as the concentration and treatment duration. But, due to the big population with AD and diabetes and the extensive usage of metformin, its effect requires a thorough exploration. Based on previous studies and our present results, we conclude that the dose and delivery time of metformin are vital for its effect. Moreover, our delivery time is before A*β* exposure, which reveals its prevention against AD. The decreased apoptosis by metformin shed a light on its application for AD strategy.

An increasing number of lines have put forward a strong correlation between JNK signaling pathway and Alzheimer's disease. A*β* increased the activation of JNK [22, 28], which is involved in the following cell injury and death. However the related mechanism is still obscure. Yenki et al. revealed that the inhibition of phosphorylated JNK decreased A*β*-induced endoplasmic reticulum stress and increased prosurvival mitochondrial proteins [29]. Mohammadi et al. found JNK inhibitor performed protection against A*β* through the reduction of autophagy and then alleviated memory deficit induced by A*β* [30]. In addition, Paquet et al. proposed JNK in human fluids may be used as one of the potential surrogate markers to appraise cell death and clinical prognosis for AD [31]. Some phosphorylation sites of JNK have been addressed. Triaca et al. reported that NGF modulated APP phosphorylation via the alteration in phosphorylation levels of the p54 JNK [32]. In particular, the phosphorylation of JNK at Thr-183/Tyr-185 is confirmed to be an early event in AD [33]. In our present study, we find metformin decreases neuronal apoptosis via suppressing the activation of JNK at the Thr-183/Tyr-185 sites, which enriched the understanding of both metformin and A*β*-induced cytotoxicity. To our knowledge, JNK-involved pathway also participates in many physiological processes in our body, limiting the application of JNK inhibitor for AD. So, as the importance of JNK in AD, metformin may be of therapeutic value for preventing AD. Besides, in the future study, the detailed interaction site between metformin and JNK deserves a thorough exploration.

Based on our current results, we can draw the following conclusions. First, JNK signaling pathway is important in A*β*-induced cytotoxicity via increasing apoptosis. Second, metformin may protect neurons through suppression of JNK signaling pathway. Third, metformin definitely protects neurons from A*β* exposure in a dose-dependent and preventive way. To aim at the clinical application of metformin for AD, we propose to find proper dose and delivery time. Although our current study only provides the first glimmer of metformin application for AD, it supports a promising strategy for AD treatment.
